# Transgene-Assisted Genetic Screen Identifies *rsd-6* and Novel Genes as Key Components of Antiviral RNA Interference in Caenorhabditis elegans

**DOI:** 10.1128/JVI.00416-18

**Published:** 2018-08-16

**Authors:** Tianyun Long, Fei Meng, Rui Lu

**Affiliations:** a Department of Biological Sciences, Louisiana State University, Baton Rouge, Louisiana, USA; University of Maryland, College Park

**Keywords:** classical RNAi, antiviral RNAi, siRNA, biased genetic screen, *rsd-6*

## Abstract

RNA interference (RNAi) is a widespread antiviral mechanism triggered by virus-produced double-stranded RNAs (dsRNAs). In Caenorhabditis elegans, antiviral RNAi involves a RIG-I-like RNA helicase, termed DRH-1 (dicer related RNA helicase 1), that is not required for classical RNAi triggered by artificial dsRNA. Currently, whether antiviral RNAi in C. elegans involves novel factors that are dispensable for classical RNAi remains an open question. To address this question, we designed and carried out a genetic screen that aims to identify novel genes involved in worm antiviral RNAi. By introducing extra copies of known antiviral RNAi genes into the reporter worms, we managed to reject alleles derived from 4 known antiviral RNAi genes, including the DRH-1 coding gene, during the screen. Our genetic screen altogether identified 25 alleles, which were assigned to 11 candidate genes and 2 known antiviral RNAi genes through genetic complementation tests. Using a mapping-by-sequencing strategy, we identified one of the candidate genes as *rsd-6*, a gene that helps maintain genome integrity through an endogenous gene-silencing pathway but was not known to be required for antiviral RNAi. More importantly, we found that two of the candidate genes are required for antiviral RNAi targeting Orsay virus, a natural viral pathogen of C. elegans, but dispensable for classical RNAi. Since *drh-1* is so far the only antiviral RNAi gene not required for classical RNAi, we believe that our genetic screen led to identification of novel worm genes that may target virus-specific features to function in RNAi.

**IMPORTANCE** In nematode worms, *drh-1* detects virus-produced double-stranded RNA (dsRNA), thereby specifically contributing to antiviral RNA silencing. To identify *drh-1*-like genes with dedicated function in antiviral RNAi, we recently carried out a genetic screen that was designed to automatically reject all alleles derived from 4 known antiviral silencing genes, including *drh-1*. Of the 11 candidate genes identified, we found two of them to be required for antiviral silencing targeting a natural viral pathogen of C. elegans but not for classical RNA silencing triggered by artificial dsRNA. We believe that these two genes are novel components of worm antiviral RNAi, considering the fact that *drh-1* is the only known antiviral RNAi gene that is dispensable for classical RNAi. This genetic screen also identified *rsd-6*, a gene that maintains genome integrity under unfavorable conditions, as a key regulator of worm antiviral silencing, demonstrating an interplay between antiviral immunity and genome integrity maintenance.

## Abstract (precis)

In nematode worms, *drh-1* detects virus-produced double-stranded RNA (dsRNA), thereby specifically contributing to antiviral RNA silencing. To identify *drh-1*-like genes with dedicated function in antiviral RNAi, we recently carried out a genetic screen that was designed to automatically reject all alleles derived from 4 known antiviral silencing genes, including *drh-1*. Of the 11 candidate genes identified, we found two of them to be required for antiviral silencing targeting a natural viral pathogen of C. elegans but not for classical RNA silencing triggered by artificial dsRNA. We believe that these two genes are novel components of worm antiviral RNAi, considering the fact that *drh-1* is the only known antiviral RNAi gene that is dispensable for classical RNAi. This genetic screen also identified *rsd-6*, a gene that maintains genome integrity under unfavorable conditions, as a key regulator of worm antiviral silencing, demonstrating an interplay between antiviral immunity and genome integrity maintenance.

## INTRODUCTION

Small interfering RNAs (siRNAs) processed from virus-derived double-stranded RNA (dsRNA) mediate potent antiviral RNA interference (RNAi) in diverse organisms (1). Mechanistic studies of antiviral RNAi have led to the identification of several key factors involved in this process. Typically, an endoribonuclease III termed Dicer processes viral dsRNA into siRNAs to initiate antiviral RNAi (2). Subsequently, an Argonaute protein, a type of endoribonuclease with an RNase H-like fold, recruits virus-derived siRNAs (vsiRNAs) as sequence guides and cleaves viral transcripts containing complementary sequence. dsRNA binding proteins also contribute to antiviral RNAi by facilitating viral dsRNA processing or vsiRNA loading into Argonaute proteins (3–5). In plants and nematodes antiviral RNAi is further amplified through the production of secondary vsiRNAs by RNA-dependent RNA polymerase (6–9). Recently, antiviral RNAi was observed in undifferentiated mammalian cells and appears to provide protection against the attack of lethal viral pathogens for suckling mice (10, 11). Since viral diseases are often the result of perturbation of host antiviral RNAi in diverse organisms (12, 13), mechanistic study of antiviral RNAi holds promise for developing novel antiviral strategies.

Orsay virus naturally infects Caenorhabditis elegans nematodes (14), providing an ideal genetic model system for the study of virus-host interactions, including antiviral RNAi. Genetic and biochemical analyses suggest that antiviral RNAi in C. elegans is initiated by the worm Dicer DCR-1, which processes viral dsRNA into primary vsiRNAs, predominately 23 nucleotides in length (4, 15, 16). Efficient processing of viral dsRNA by DCR-1 requires a dsRNA-binding protein termed RDE-4 (RNAi defective 4) (4, 5, 17, 18). The primary vsiRNAs are then loaded into RDE-1, one of the worm Argonaute (Ago) proteins that has slicer activity (4, 17–19). Instead of cleaving viral transcripts with matching sequence, RDE-1 loaded with primary vsiRNA activates, probably with help from cofactors, RRF-1, a worm RNA-dependent RNA polymerase (RdRp) (19, 20). Subsequently, activated RRF-1 synthesizes secondary siRNAs in a DCR-1-independent manner (6, 8, 21–23). Unlike primary vsiRNAs produced by DCR-1, secondary vsiRNAs are single-stranded RNA molecules of 22 nucleotides and carry a triphosphate group at the 5′ end (22–24).

Antiviral RNAi in C. elegans also requires DRH-1 (Dicer-like RNA helicase 1) and DRH-3, two RIG-I-like RNA helicases (RLHs) that are not conserved in fungi, plants, or insects (16, 25). Previously we found that the C-terminal regulatory domain of human RIG-I protein, which contributes to virus sensing in mammalian innate immunity (26–29), can functionally replace the corresponding domain in DRH-1, suggesting a role of DRH-1 in virus sensing (8). Consistent with this, virus-derived vsiRNAs were found to be significantly reduced in *drh-1* mutants (6, 8). Currently, it remains largely unknown whether DRH-1-mediated virus sensing involves additional factors and how the function of DRH-1 is regulated in response to virus invasion. Although sharing sequence homology and domain structure with DRH-1, DRH-3 appears to function downstream of DRH-1 and is required for the production of secondary vsiRNA (6, 8). DRH-3 also plays an essential role in worm development (30). Currently exactly what DRH-3 does in antiviral RNAi remains largely unknown.

RSD-2 is another key component of worm antiviral RNAi and appears to be conserved only in the nematode kingdom (16, 31, 32). Previously we have shown that RSD-2 contributes to both RDE-4-dependent and RDE-4-independent antiviral RNAi (31). Consistent with a prior study on classical RNAi (33), we found that vsiRNAs can by readily detected by Northern blotting in *rsd-2* mutants with a size distribution similar to that in *rde-1* and *rrf-1* mutants, suggesting that RSD-2 is dispensable for the biogenesis of primary vsiRNAs and contributes to the amplification of antiviral RNAi by facilitating the production of secondary vsiRNAs (31). Recently, a Vasa ATPase-related protein, termed RDE-12, was also found to contribute to antiviral RNAi, probably by enabling the production of secondary vsiRNA (34).

Previously we have shown that high-level viral replication in *drh-1* mutants led to the production of vsiRNAs at low levels (8). This observation, together with a recent report, suggests a *drh-1*-independent mechanism for the production of vsiRNAs in C. elegans (35). Notably, these vsiRNAs are capable of mediating potent silencing of a homologous transgene (8). Similarly, silencing of transgenes but not homologous virus can also be triggered in *drh-1* mutants when RNAi is induced using artificial dsRNA (8). These findings together suggest that viruses are more resistant to RNAi than homologous cellular transcripts, and as such additional genes may be required for antiviral RNAi. Apparently, these antivirus-specific genes can only be identified in genetic screens where replicating viruses are used as reporters of loss of RNAi.

Orsay virus is a plus-strand RNA virus with a bipartite genome that resembles nodavirus in terms of structure and sequence homology. Orsay virus only infects intestine cells of C. elegans, and such a tissue-specific infection pattern remains unchanged when the virus was delivered into wild-type worms or RNAi-defective mutants as a transgene known to be active in nonintestinal cells (36). These observations suggest that there is a receptor- and RNAi-independent mechanism that restricts systemic spreading of Orsay virus in C. elegans. Orsay virus replicates at low levels in wild-type N2 worms but accumulates to high levels in RNAi-defective mutants or in the presence of a functional RNAi suppressor (6, 8, 31, 37). These observations, together with the fact that Orsay virus was originally isolated from a worm isolate naturally defective in antiviral RNAi, suggest that Orsay virus has very weak, if any, activity in RNAi suppression (14). Currently a modified Orsay virus derivative that would allow for the visualization of loss of antiviral RNAi in C. elegans is still lacking. Thus, genetic screens that aim to identify novel antiviral RNAi genes through genetic screen will need to seek an alternative model virus as a reporter for loss of antiviral RNAi.

To identify novel genes with dedicated function in antiviral RNAi, we have recently carried out a large-scale genetic screen that utilized a flock house virus (FHV) replicon as the loss-of-RNAi reporter (16). By integrating extra copies of four known antiviral RNAi genes into the reporter transgene array, we expected to automatically reject genetic alleles derived from those four genes during genetic screening. Upon completing such a biased genetic screen, we isolated 25 genetic alleles that were assigned to 11 candidate genes and two known RNAi genes. Most importantly, we found that two of the candidate genes are required for antiviral RNAi targeting Orsay virus but dispensable for classic RNAi. Since *drh-1* alleles have been excluded during the screen, we believe that these two candidate genes are a novel requirement of worm antiviral RNAi. Using a mapping-by-sequencing strategy, we also identified one candidate gene as *rsd-6. rsd-6* plays a role in endogenous gene silencing that helps maintain genome integrity but was not known to be required for antiviral RNAi. Our study thus revealed an interplay between antiviral immunity and an endogenous gene silencing pathway that maintains genome integrity.

## RESULTS

### The design of a biased genetic screen for the identification of C. elegans genes required for antiviral RNAi.

**FIG 1:**
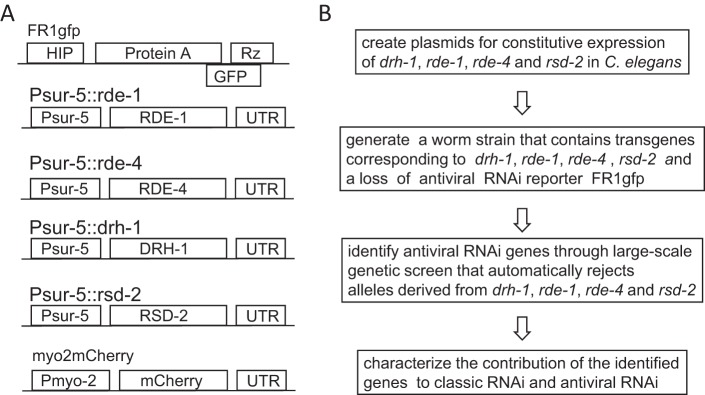
Design of a biased genetic screen for the identification of novel C. elegans genes involved in antiviral RNAi. (A) Schematic structure of plasmid constructs to be used to create the reporter transgene array for biased genetic screen. HIP, heat-inducible promoter; protein A, the replicase of Flock house virus; Rz, self-cleaving ribozyme sequence derived from hepatitis D virus, which functions to remove all nonviral sequence at the 3′ end of the FR1gfp primary transcripts; GFP, the coding sequence of enhanced green fluorescence protein; Psur-5, the promoter of the endogenous gene *sur*-5; UTR, the 3′-end untranslated region of *unc-54*; Pmyo-2, the myo-2 promoter which directs gene expression in pharyngeal muscles. (B) The workflow for identification of worm genes required for antiviral RNAi.

FHV is a member of the *Nodaviridae* family. Although not a pathogen of nematode worms, when delivered as a transgene, FHV replicates and triggers potent antiviral RNAi in C. elegans (16), making it an alternative model virus for the study of RNAi-mediated virus-host interaction in C. elegans (8, 17, 31, 37–39). Most importantly, an FHV RNA1 derivative, named FR1gfp, has been successfully developed as a reporter for loss of antiviral RNAi in C. elegans (Fig. 1A) (16). FR1gfp features an enhanced green fluorescent protein (eGFP) coding sequence in place of B2 coding sequence and produces bright green fluorescence in worm mutants defective in RNAi. Thus, FR1gfp combined with a large-scale genetic screen will allow for the identification of novel genes involved in antiviral RNAi.

So far, 10 C. elegans genes, including *dcr-1*, *drh-1*, *drh-3*, *mut-7*, *rde-1*, *rde-2*, *rde-4*, *rde-12*, *rrf-1*, and *rsd-2*, have been implicated in antiviral RNAi through genetic analysis (4, 6, 8, 16–18, 31, 34). Since most of these genes, with the exception of *dcr-1*, *drh-3*, and *mut-7*, are dispensable for worm development, a conventional forward genetic screen that aims to identify novel genes involved in antiviral RNAi may inevitably and repetitively pick up loss-of-function alleles derived from some of these genes.

In C. elegans, gonad microinjection of target gene constructs often leads to the formation of large transgene arrays that contain many copies of target genes (40). Since the formation of those large transgene arrays does not rely on the sequence homology shared between the injected genes, it is possible to generate a large transgene array that contains many different transgenes. This observation suggests that we can codeliver the FR1gfp reporter and several transgenes corresponding to known antiviral RNAi genes into the worm strain to be used for the genetic screen. Since the chance to simultaneously mutate both the transgenes and corresponding endogenous genes will be extremely low, considering the fact that most of the transgenes will be delivered in multiple copies, genetic screens utilizing this novel worm strain will automatically reject null alleles derived from the known antiviral RNAi genes chosen to be excluded during the screen. Therefore, to better understand worm antiviral RNAi, we have developed an experimental strategy, as shown in Fig. 1, for identifying and characterizing worm genes with dedicated function in antiviral RNAi. In this strategy, a reporter transgene array is created to contain both a heat-inducible FR1gfp transgene and transgenes constitutively expressing four of the known antiviral RNAi genes, *drh-1*, *rde-1*, *rde-4*, and *rsd-2* (Fig. 1A). The FR1gfp transgene serves as a loss-of-antiviral-RNAi reporter, whereas the other 4 transgenes ensure that no loss-of-function alleles corresponding to these genes will be isolated during the genetic screens. Following the genetic screen the candidate mutants will be subjected to classical RNAi test to see whether they specifically contribute to antiviral RNAi (Fig. 1B).

### Generation of a reporter transgene array for biased genetic screen.

**FIG 2:**
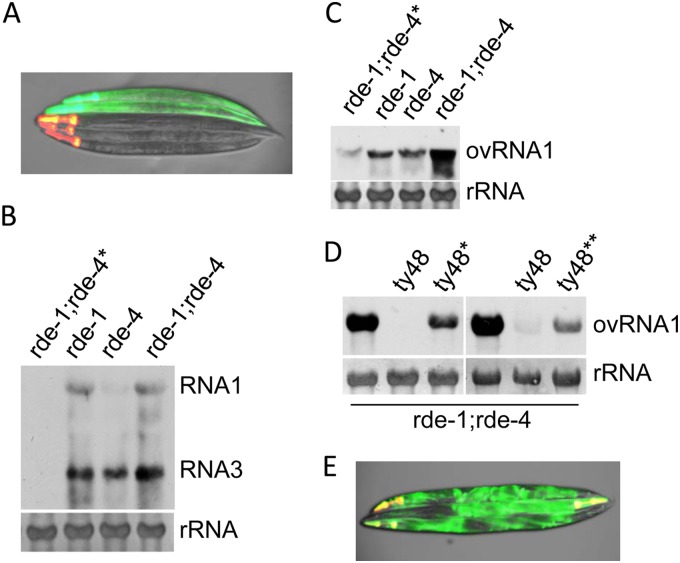
Generation of reporter transgene array ty48. (A) Coinjection of the plasmid constructs shown in Fig. 1A restored antiviral RNAi in *rde-1*;*rde-4* double mutants that contain an FR1gfp transgene. Shown here is the visualization of green fluorescence in the *rde-1*;*rde-4* double mutants that contain extrachromosomal arrays formed by the injected plasmids 48 h after heat induction. The image was produced by merging images recorded under red and green fluorescence using the same exposure. (B) Accumulation of FR1gfp transcripts in single and double mutants corresponding to *rde-1* and *rde-4* as indicated. An asterisk indicates worms that carry the ty48 transgene array. The FR1gfp transcripts were detected by Northern blotting, for which the probe was prepared using GFP coding sequence; RNA1, FR1gfp genomic RNA; RNA3, FR1gfp subgenomic RNA. Methylene blue-stained rRNA serves as an equal loading control. (C) Northern blot detection of Orsay virus RNA1, ovRNA1, in single and double mutants corresponding to *rde-1* and *rde-4* as indicated. An asterisk indicates worms that carry the ty48 transgene array. Orsay virus RNA1 cDNA was used to prepare probe for the detection of Orsay virus RNA1 through Northern blotting. (D) Accumulation of Orsay virus RNA1 in response to *rde-1* or *rde-4* dsRNA feeding in *rde-1*;*rde-4* double mutants with or without the ty48 transgene array. An asterisk indicates worms that were fed with E. coli food expressing *rde-1* dsRNA. A double asterisk indicates worms that were fed with E. coli food expressing *rde-4* dsRNA. (E) The FR1gfp transgene in the transgene array ty48 is functional. Shown here is the visualization of green fluorescence in the wild-type N2 worms carrying the ty48 transgene array 48 h after heat induction. The worms have been fed using E. coli food expressing *rde-4* dsRNA.

To generate a worm strain that contains both a heat-inducible FR1gfp transgene and transgenes corresponding to *drh-1*, *rde-1*, *rde-4*, and *rsd-2*, we combined six plasmid constructs, as shown in Fig. 1A, and injected them into *rde-1*;*rde-4* double mutants that already contain a heat-inducible FR1gfp transgene. This microinjection led to the production of 5 transgenic lines that contain transmissible extrachromosomal arrays. To find out whether the *rde-1* and *rde-4* transgenes in these extrachromosomal arrays are functional, we checked the expression of green fluorescence in the transgenic animals after heat induction. Although a few worms occasionally showed green fluorescence in pharyngeal tissue, none of the transgenic animals, marked by red fluorescence in the head, produced whole-body green fluorescence (Fig. 2A), confirming that both *rde-1* and *rde-4* transgenes in those extrachromosomal transgene arrays are functional. To generate a reporter strain for genetic screens, we treated one of the transgenic lines with gamma ray irradiation and screened the F2 worms for integrated transgene arrays. This led to the identification of 3 integrated transgene arrays in total. By checking the production of green fluorescence in response to heat induction, we confirmed that all of these integrated transgene arrays contain functional *rde-1* and *rde-4* transgenes. We chose one of the integrated transgene arrays, termed ty48, for further characterization mainly because worms carrying this transgene array are free of any developmental defects.

After introducing the ty48 transgene array into nontransgenic *rde-1*;*rde-4* double mutants through outcross, we performed Northern blot analyses to detect the replication of FR1gfp and Orsay virus in the resulting worm strain. As shown in Fig. 2B and C, neither of the viruses was able to replicate at high level, and accordingly, no whole-body green fluorescence was observed after heat induction (data not shown). However, when *rde-1*;*rde-4* double mutants carrying the ty48 transgene array were fed with E. coli food expressing either *rde-1* or *rde-4* dsRNA, enhanced Orsay virus replication was detected through Northern blot analyses (Fig. 2D), suggesting that the restoration of antiviral RNAi in the double mutants is indeed due to the introduction of functional *rde-1* and *rde-4* transgenes. As shown in Fig. 2E, *rde-4* dsRNA feeding led to the production of whole-body green fluorescence in the double mutants after heat induction, suggesting that the FR1gfp transgene was successfully integrated into the ty48 transgene array.

### Both *drh-1* and *rsd-2* transgenes are functional in the ty48 transgene array.

**FIG 3:**
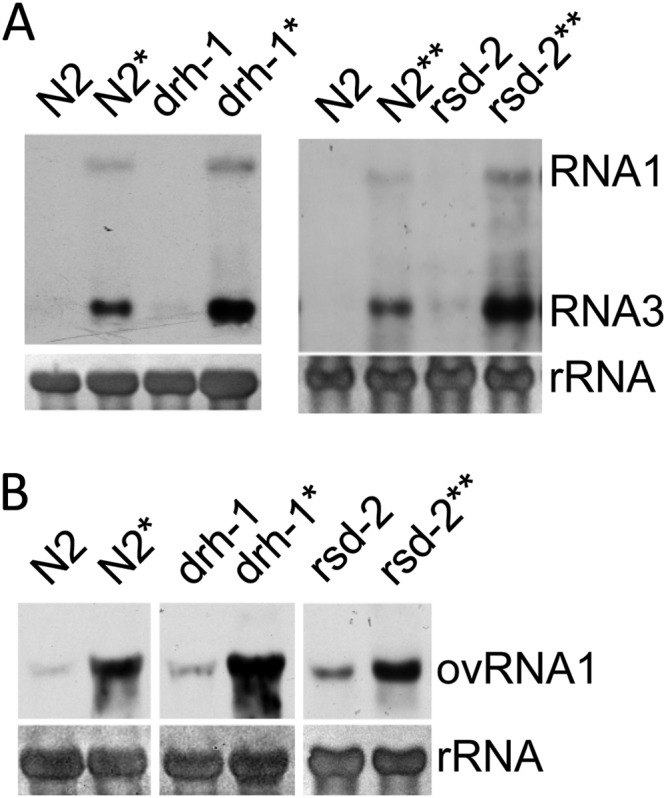
Both *drh-1* and *rsd-2* transgenes are functional in the ty48 transgene array. (A) Northern blot detection of FR1gfp replication in transgenic worm strains, as indicated, that contain the ty48 transgene array after heat induction. See the legend to Fig. 2B for experimental details. An asterisk indicates worms that were fed with E. coli food expressing *drh-1* dsRNA. A double asterisk indicates worms that were fed with E. coli food expressing *rsd-2* dsRNA. (B) Accumulation of Orsay virus RNA1 in *drh-1* and *rsd-2* mutants and wild-type N2 worms that carry the ty48 transgene array. An asterisk indicates worms that were fed with E. coli food expressing *drh-1* dsRNA. A double asterisk indicates worms that were fed with E. coli food expressing *rsd-2* dsRNA. See the legend to Fig. 2C for experimental details.

To find out whether *drh-1* and *rsd-2* transgenes have also been successfully integrated in the transgene array ty48, we transferred ty48 into *drh-1* and *rsd-2* mutants through outcross and checked FR1gfp replication in the resulting worm strains after heat induction. We found that, as shown in Fig. 3A, the ty48 transgene array successfully restored antiviral RNAi in *drh-1* and *rsd-2* mutants, leading to the suppression of FR1gfp replication. Moreover, no whole-body green fluorescence was observed in mutant worms containing the ty48 transgene array (data not shown). We found that the replication of Orsay virus was also suppressed to a level comparable to that in wild-type N2 worms containing the same transgene array (Fig. 3B). Apparently, the restoration of antiviral RNAi in both mutants can only be ascribed to the introduction of the ty48 transgene array, since the replication of both FR1gfp and Orsay virus was restored when the expression of *drh-1* and *rsd-2*, from both the endogenous genes and transgenes, were suppressed through feeding RNAi (Fig. 3A and B). These results together suggest that both *drh-1* and *rsd-2* transgenes were successfully integrated into the ty48 transgene array.

### Antiviral RNAi remains defective in *drh-3* and *rrf-1* mutants containing the ty48 transgene array.

**FIG 4:**
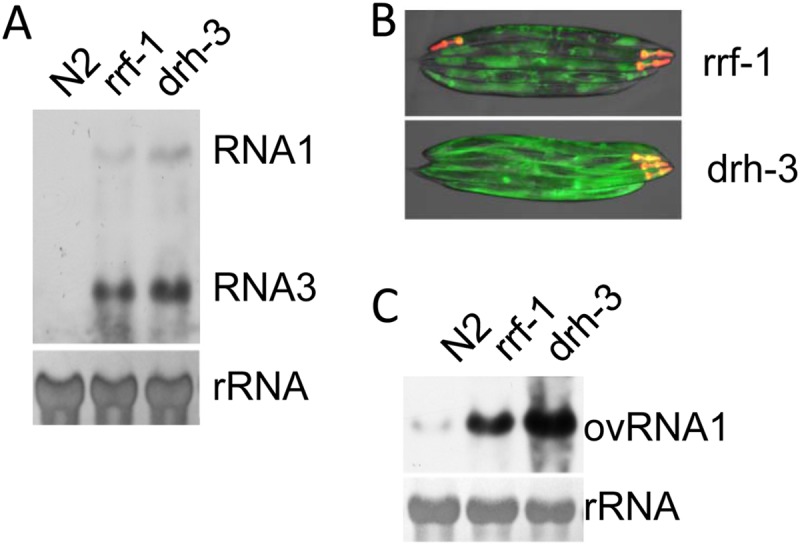
Antiviral RNAi remains defective in *rrf-1* and *drh-3* mutants containing the ty48 transgene array. (A) Northern blot detection of FR1gfp replication in *rrf-1* and *drh-3* mutants, as indicated, that contain the ty48 transgene array. See the legend to Fig. 2B for experimental details. (B) Visualization of green fluorescence in *rrf-1* and *drh-3* mutants, as indicated, carrying the ty48 transgene array 48 h after heat induction. These images were produced by merging images recorded using the same exposure and at the same time. (C) Accumulation of Orsay virus RNA1 in *rrf-1* and *drh-3* mutants that carry the ty48 transgene array.

The RNA-dependent RNA polymerase *RRF-1* contributes to antiviral RNAi by synthesizing the secondary vsiRNAs (5, 15, 20, 41, 42). Although secondary vsiRNAs become undetectable in the absence of DRH-3, it is possible that *DRH-3* does more than facilitate the production of secondary vsiRNAs, as both FHV and Orsay virus accumulate to higher levels in *drh-3* mutants than that in *rrf-1* mutants (8). To find out whether the ty48 transgene array is sensitive enough to pick up genetic mutations that disrupt antiviral RNAi, we introduced the ty48 transgene array into *drh-3* and *rrf-1* mutants and checked the replication of both FR1gfp and Orsay virus in the resulting worm strains. We found that, as shown in Fig. 4A and B, FR1gfp replicated to high levels in both *drh-3* and *rrf-1* upon heat induction, leading to the production of green fluorescence in the resulting worm strains. Consistent with this, enhanced Orsay virus replication was also detected in both mutant strains compared to that in wild-type N2 worms containing the same transgene array (Fig. 4C). Importantly, we found that both FR1gfp and Orsay virus accumulated to higher levels in *drh-3* mutants than that in *rrf-1* mutants, and accordingly *drh-3* mutants produced green fluorescence with higher intensity than that from *rrf-1* mutants (Fig. 4B). These results together suggest that we have successfully developed a transgene array that can be used as a reporter for the identification of loss of antiviral RNAi mutations in large-scale genetic screens. Since the heat-inducible promoter used in this study is active in most worm cells and the FR1gfp replicon is not capable of cell-cell movement, a genetic screen utilizing ty48 as a reporter is expected to allow us to identify antiviral RNAi genes that function in a cell-autonomous manner.

### Identification of novel antiviral RNAi genes through large-scale genetic screen.

To identify novel genes involved in worm antiviral RNAi, we carried out a large-scale genetic screen using N2 worms carrying the ty48 transgene array as a reporter. We used ethyl methane sulfonate (EMS) as a mutagen to introduce random mutations into the reporter worms by following a standard protocol. To reduce allele duplicates we bleached F1 worms and collected the eggs for screening. After heat induction we picked up F2 worms that produced green fluorescence at a level higher than or equal to that in *rrf-1* mutants carrying the same ty48 transgene array for further analysis. This screen led to the identification of 25 alleles that compromised antiviral RNAi. To find out whether any of these alleles were temperature-sensitive alleles, we checked the fertility of the corresponding worms when they were maintained at 25°C. This test identified *1026f*, *1027e*, *1103a*, and *1026g* as temperature-sensitive alleles (Table 1). Worms carrying these alleles also exhibited developmental defects such as reduced brood size. The fertility of the rest of the 21 mutants was not significantly affected by elevated temperature. However, worms carrying *1026a*, *1031a*, *1031f*, and *1105a* alleles also exhibited reduced brood size, whereas worms with *1030c* produced dead eggs and more males than wild-type N2 worms under normal growth conditions. Together, these observations suggest that some of the identified genes play important roles in worm development.

**TABLE 1:** Loss of antiviral RNAi alleles isolated in the biased genetic screen

Candidate gene	Allele	Strength of green fluorescence^*a*^	Temp sensitivity^*b*^	Developmental defect
*asd-1*	*1026f*	++++	+	Reduced brood size
	*1027e*	+++	+	Reduced brood size
	*1103a*	++++	+	Reduced brood size
	*1026g*	++++	+	Reduced brood size
*asd-2*	*1026b*	++++	−	−
	*1028g*	++++	−	−
	*1029e*	++++	−	−
*asd-3*	*1029c*	++++	−	−
	*1031e*	++++	−	−
	*1102b*	+++	−	−
*asd-4*	*1025b*	+++	−	−
	*1028f*	++++	−	−
	*1029b*	++++	−	−
*asd-5*	*1026a*	++++	−	Reduced brood size
	*1031a*	++++	−	Reduced brood size
	*1031f*	++++	−	Reduced brood size
*asd-6* (*rde-3*)	*1031b*	++++	−	−
*asd-7*	*1028j*	++++	−	−
*asd-8*	*1030d*	+++	−	−
*asd-9*	*1026d*	+++	−	−
*asd-10*	*1105a*	+++	−	Reduced brood size
*asd-11*	*1030c*	+++	−	High frequency of males
*asd-12*	*1026c*	+++	−	−
*asd-13* (*rrf-1*)	*1025a*	+++	−	−
	*1026e*	+++	−	−

a The GFP expression level in *drh-3* and *rrf-1* mutants was used as a reference. Candidate mutants that produce green fluorescence at a level higher than or equal to that in *rrf-1* mutants carrying the same ty48 transgene array are marked as +++. Candidate mutants producing green fluorescence at a level higher than or equal to that in *drh-3* mutants are marked as ++++.

b Candidate mutants that are sterile at 25°C are defined as temperature-sensitive mutants.

**FIG 5:**
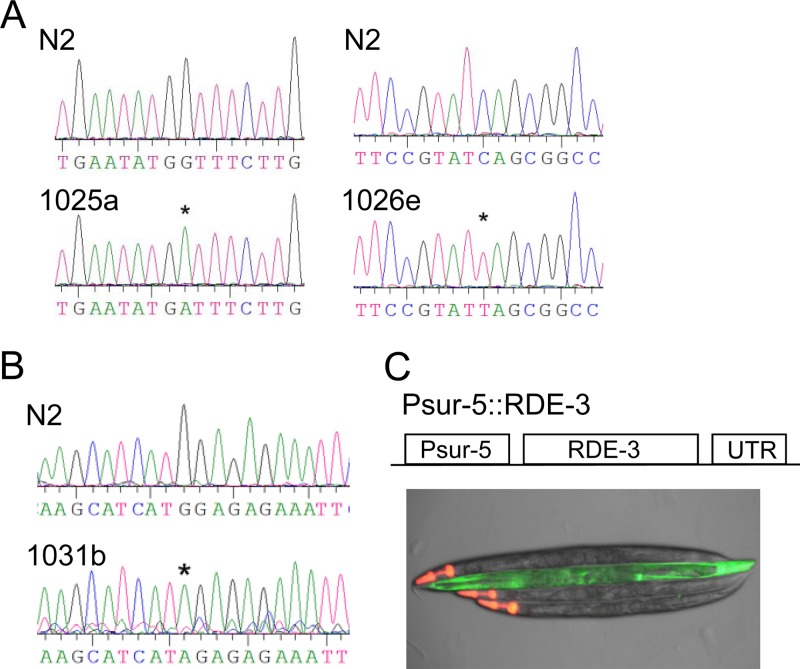
Identification of *rrf-1* and *rde-3* alleles that are defective in antiviral RNAi. (A) Identification of point mutations in *rrf-1* coding sequence that disrupt *rrf-1* function in antiviral RNAi. Shown here is the *rrf-1* cDNA sequencing results for *rrf-1* alleles 1025a and 1026e. The *rrf-1* coding sequence in wild-type worm N2 was sequenced as a reference. (B) Point mutations in *rde-3* allele 1031b that disrupt antiviral RNAi. The *rde-3* coding sequence in wild-type worm N2 was sequenced as a reference. (C) Ectopic expression of wild-type *rde-3* restored antiviral RNAi in worm mutants containing the 1031b allele. (Upper) Structure of plasmid construct that expresses wild-type *rde-3*. (Lower) Visualization of antiviral RNAi in worms containing the 1031b allele. Worms marked with red fluorescence in the head region contain a transgene that expresses wild-type *rde-3*.

To determine the dominance of the identified alleles, we performed crosses between the identified mutants and wild-type N2 worms and checked the occurrence of loss of antiviral RNAi phenotype in the F2 generations. This test confirmed that all of the identified alleles were recessive (data not shown). Through genetic complementation tests we successfully assigned the identified alleles to 13 distinct genes, here referred to as antiviral silencing-defective (*asd*) genes (Table 1). Interestingly, all temperature-sensitive alleles, which also exhibit similar developmental defects, were assigned to a single gene, *asd-1*. To find out whether any of our candidate genes are known antiviral RNAi genes, we performed extensive genetic complementation tests between our candidate worms and worm strains that contain loss-of-function alleles corresponding to *drh-1*, *drh-3*, *rde-1*, *rde-2*, *rde-3*, *rde-4*, *rde-10*, *rde-11*, *rrf-1*, and *rsd-2*. These tests not only identified *asd-6* and *asd-13* as *rde-3* and *rrf-1* (Table 1) but also confirmed that none of the remaining 11 genes are *drh-1*, *rde-1*, *rde-4*, *rsd-2*, or *drh-3* (Table 1). Through cDNA sequencing we found that the *1025a* allele of *rrf-1* contains a point mutation that caused an alteration from glycine to aspartic acid at position 540 of RRF-1, whereas the *1026e* allele of *rrf-1* carried a premature stop codon after the first 96 codons (Fig. 5A). The *1031b* allele of *rde-3* contains a premature stop codon, causing a deletion of 350 amino acids (aa) at the C terminus of RDE-3 protein, which is 441 aa in total (Fig. 5B). We believe that *1026e* and *1031b* are null alleles for *rrf-1* and *rde-3*, respectively. To reconfirm that it is the mutation in the *1031b* allele that caused the loss of antiviral RNAi, we injected the mutant worms with plasmid expressing wild-type RDE-3. As expected, ectopic expression of wild-type RDE-3 indeed restored the antiviral silencing (Fig. 5C). In addition to *rrf-1*, multiple alleles were also identified for *asd-1*, *asd-2*, *asd-3*, *asd-4*, and *asd-5* (Table 1). We noticed that alleles for most of these 5 candidate genes appeared to have been isolated from different batches of mutagenized worms, suggesting that they are independent alleles. The identification of *rde-3* and *rrf-1* alleles together with the fact that distinct alleles were also picked up for several other candidate genes suggests that our biased genetic screen had a comprehensive coverage on antiviral RNAi genes for which the antiviral function can be compromised without sacrificing worm viability.

### Functional characterization of the identified candidate genes.

**FIG 6:**
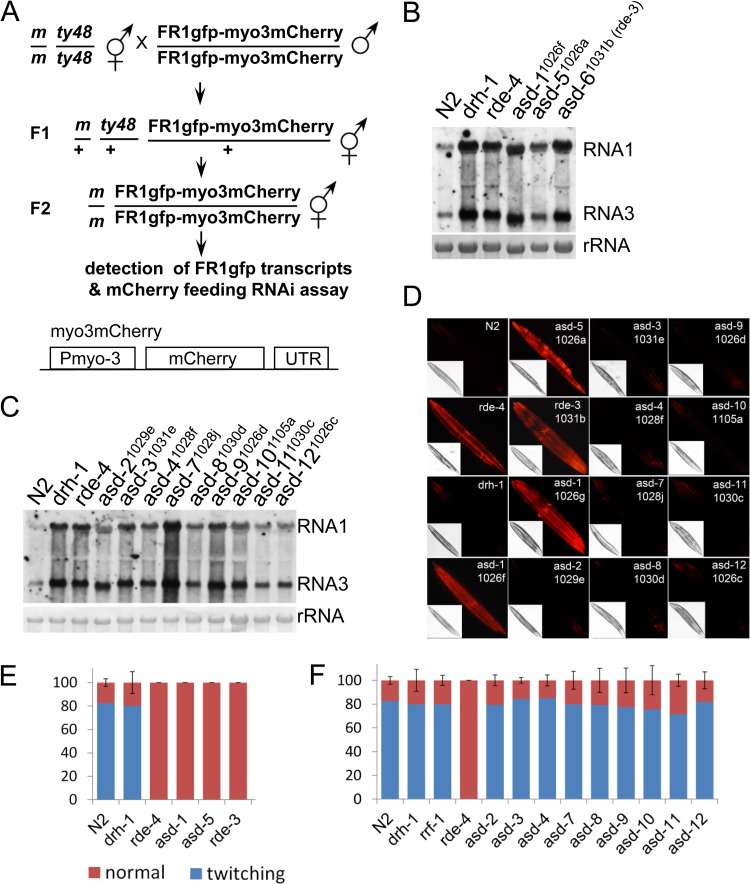
Function characterization of the candidate genes. (A) Experimental design for the introduction of candidate alleles into worm strain LR11. m, loss-of-function alleles identified in the biased genetic screen. Pmyo-3, myo-3 promoter that is constitutively active in body wall muscle. (B) Northern blot detection of FR1gfp replication in LR11 worms that contain genetic alleles corresponding to *asd-1*, *asd-5*, and *rde-3*. The replication of FR1gfp was also detected in wild-type N2 worms and *drh-1* and *rde-4* mutants as references. (C) Northern blot detection of FR1gfp replication in LR11 worms that contain genetic alleles corresponding to *asd-2*, *asd-3*, *asd-4*, *asd-7*, *asd-8*, *asd-9*, *asd-10*, *asd-11*, and *asd-12*. (D) Visualization of mCherry fluorescence in LR11 worms that contain genetic alleles corresponding to candidate alleles, as indicated within each set of images. All worms were fed using E. coli food expressing dsRNA derived from mCherry coding sequence. The inset images were taken under white light. All images were taken using the same exposure. (E and F) *unc-22* RNAi phenotype observed in LR11 worms containing genetic alleles, as indicated in panels B and C, corresponding to candidate genes. *unc-22* dsRNA was injected at a concentration of 100 ng/μl. Shown here are the percentages of twitching F1 progenies of the injected worms collected between 8 and 32 h postinjection. The error bars indicate standard deviations for the twitching phenotype.

With a replicating virus as the trigger and target of RNAi, our genetic screen was expected to identify genes with specific function in antiviral RNAi but not in classical RNAi. To determine whether any of the candidate genes contributed specifically to antiviral RNAi, we tested whether their function in RNAi was triggered by dsRNA ingestion. We did not use the original mutants for this test, considering that some of the random mutations in the original mutants may have adverse effects on feeding RNAi. Our test began with the introduction, through outcross, of the representative allele for each of the 11 *asd* genes into a transgenic worm strain, termed LR11 (Fig. 6A, upper). LR11 worms contain two physically linked transgenes, a heat-inducible FR1gfp transgene and an mCherry transgene driven by the *myo-3* promoter (Fig. 6A, lower). The FR1gfp transgene produces green fluorescence upon heat induction when antiviral RNAi is defective, whereas the mCherry transgene directs constitutive expression of mCherry in body wall muscle and serves as the target of feeding RNAi. After homozygous alleles corresponding to the identified genes were introduced into LR11 worms, as confirmed by visualization of green fluorescence after heat induction, we checked RNAi response in the resulting worms upon mCherry dsRNA ingestion. We reasoned that mCherry dsRNA ingestion would lead to the silencing of mCherry expression in body wall muscle if the allele specifically disrupted antiviral RNAi but not classical RNAi. In contrast, no mCherry silencing should occur if the tested allele disrupts both classical RNAi and antiviral RNAi. We found that, as shown in Fig. 6B, C, and D, although enhanced FR1gfp replication was detected at comparable levels, mCherry silencing was observed for alleles corresponding to *asd*-2, *asd-3*, *asd-4*, *asd-7*, *asd-8*, *asd-9*, *asd-10*, *asd-11*, and *asd-12* but not for alleles corresponding *asd-1*, *asd-5*, and *asd-6* (*rde-3*). As a reconfirmation, we subjected the same set of worms to feeding RNAi tests in which the endogenous genes *skn-1* and *unc-22* were chosen as the targets of RNAi. Again, as shown in Table 2, penetrating RNAi phenotypes, manifested as dead eggs and twitching progenies, were observed for alleles corresponding to *asd-2*, *asd-3*, *asd-4*, *asd-7*, *asd-8*, *asd-9*, *asd-10*, *asd-11*, and *asd-12* but not for *asd-1*, *asd-5*, and *asd-6* (*rde-3*). To rule out the possibility that the differential requirement of the identified genes in classical RNAi is an artifact associated with dsRNA feeding, we tested the same set of worm mutants in RNAi response triggered by *unc-22* dsRNA microinjection. As shown in Fig. 6E and F, injection of *unc-22* dsRNA at a concentration of 100 ng/μl induced severe twitching phenotypes in the progenies of injected N2 worms and LR11 worms carrying *drh-1* null allele *tm1329* and *rrf-1* null allele *ok589* but not in LR11 worms carrying *rde-4* null allele *ne337*. The same treatment induced a strong twitching phenotype for progeny worms corresponding to *asd-2*, *asd-3*, *asd-4*, *asd-7*, *asd-8*, *asd-9*, *asd-10*, *asd-11*, and *asd-12* but not for progeny worms corresponding to the rest of the *asd* genes. These observations together suggest that nine of our candidate genes mainly function in antiviral silencing but not classical RNAi.

**TABLE 2:** Sensitivity of the identified mutants to feeding RNAi targeting *skn-1* and *unc-22*^*a*^

Candidate gene	Associated alleles	Sensitivity to *skn-1* dsRNA feeding	Sensitivity to *unc-22* dsRNA feeding
*asd-1*	*1026f*	−	−
	*1027e*	−	−
	*1103a*	−	−
	*1026g*	−	−
*asd-2*	*1026b*	+	+
	*1029e*	+	+
	*1028g*	+	+
*asd-3*	*1029c*	+	+
	*1031e*	+	+
	*1102b*	+	+
*asd-4*	*1025b*	+	+
	*1028f*	+	+
	*1029b*	+	+
*asd-5*	*1026a*	−	−
	*1031a*	−	−
	*1031f*	−	−
*asd-6* (*rde-3*)	*1031b*	−	−
*asd-7*	*1028j*	+	+
*asd-8*	*1030d*	+	+
*asd-9*	*1026d*	+	+
*asd-10*	*1105a*	+	+
*asd-11*	*1030c*	+	+
*asd-12*	*1026c*	+	+

a The sensitivity to *skn-1* dsRNA feeding was recorded as whether the eggs laid by the treat worms can hatch. The sensitivity to *unc-22* dsRNA feeding was recorded as whether the progenies of the treated worms display twitching phenotype. −, not sensitive; +, sensitive.

### Identification of *asd-5* as *rsd-6* through whole-genome sequencing.

**FIG 7:**
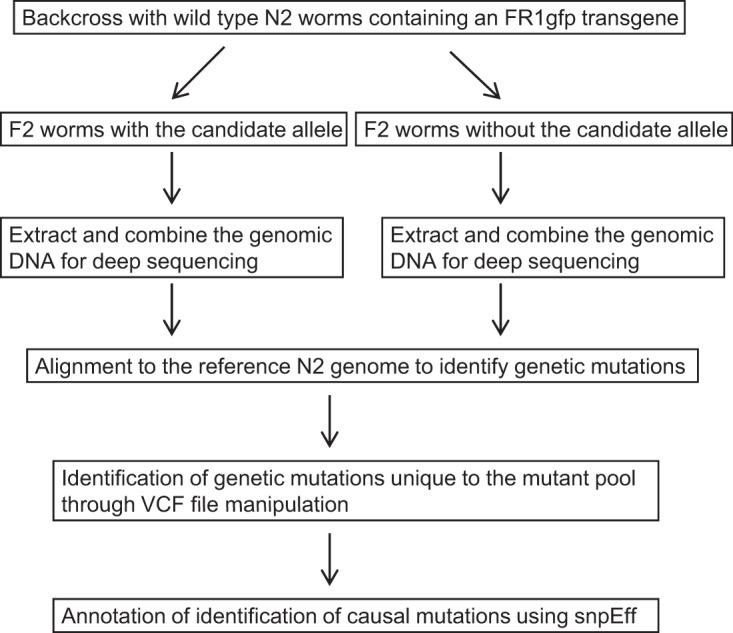
Schematic presentation of the workflow for the identification of candidate alleles using the mapping-by-sequencing strategy. VCF, variant call format. snpEff, a toolbox for genomic variant annotations and functional effect prediction.

**FIG 8:**
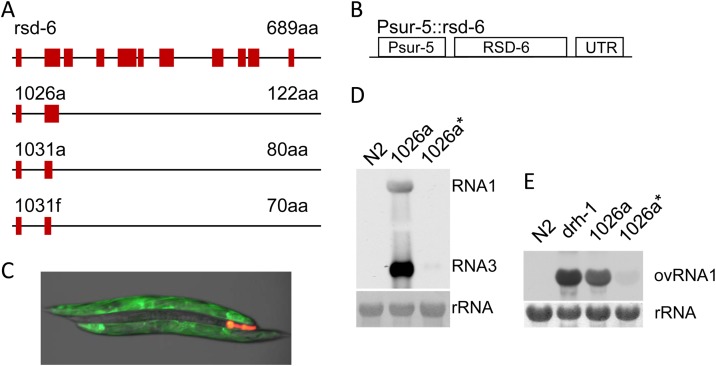
Identification of *rsd-6* as key component of antiviral RNAi in C. elegans. (A) Schematic gene structures for wild-type *rsd-6* and *rsd-6* alleles isolated in the genetic screen. Exons are shown as solid boxes, whereas the lines indicate introns. The putative protein sizes from conceptual translation are indicated for each allele. (B) Schematic structure of the plasmid construct that was used for constitutive expression of wild-type *rsd-2* gene in transgenic worms. RSD-6, the open reading frame of wild-type *rsd-6*. (C) Ectopic expression of wild-type *rsd-6* restored antiviral RNAi in worm mutants containing the *1026a* alleles. Shown here is the visualization of green fluorescence in the *1026a* mutants that have been injected with plasmid construct Psur-5::RSD-6. The transgenic worm is marked by red fluorescence in the head region. (D) Constitutive overexpression of wild-type *rsd-6* restored antiviral RNAi in worm mutants containing the *1026a* alleles. Shown here is the detection of FR1gfp genomic and subgenomic RNA accumulation after heat induction. An asterisk indicates 1026a mutants that contain integrated transgene derived from the plasmid construct Psur-5::RSD-6. (E) Same as panel D, except that the replication of Orsay virus RNA1 was detected through Northern blotting. The replication of Orsay virus RNA1 in *drh-1* loss-of-function mutants was detected as the reference.

Worms containing *1026a*, *1031a*, and *1031f* alleles lay fewer eggs than wild-type worms but are generally healthy and exhibit normal mobility. To gain insight into the genetic identity of *asd-5*, we used the mapping-by-sequencing strategy to identify one of its alleles, *1026a*. Using an in-house-developed workflow, as illustrated in Fig. 7, we mapped the *1026a* allele to a point mutation in worm gene *rsd-6*. By sequencing *rsd-6* cDNA we confirmed that a C-to-T point mutation at position 367 has caused a premature stop codon in *rsd-6* coding sequence in the *1026a* worms, and such a mutation led to the deletion of the last 567 aa of RSD-6 protein, which is 689 aa in length (Fig. 8A). Importantly, point mutations that caused premature stop codons in *rsd-6* coding sequence were also identified for another two *asd-5* alleles, *1031a* (C-to-T change at position 241) and *1031f* (G-to-A change at position 213). We believe that all of these new *rsd-6* alleles are null alleles, considering the large deletions in the RSD-6 C-terminal half for these alleles (Fig. 8A). To reconfirm that *asd-5* is indeed *rsd-6*, we created an RSD-6-expressing construct (Fig. 8B) and used it to inject worms containing the *1026a* allele. We reasoned that if the loss of antiviral RNAi in *1026a* worms is caused by genetic mutations in *rsd-6*, ectopic expression of wild-type *rsd-6* will restore antiviral RNAi in *1026a* worms. As shown in Fig. 8C, antiviral RNAi was indeed restored in all *1026a* worms that carry the extrachromosomal arrays formed by the Psur-5::rsd-6 transgene (Fig. 8C, compare worms with red fluorescence in the head to worms without). Consistent with this, FHV and Orsay virus replication was significantly reduced in *1026a* worms that contain the integrated transgene corresponding to the injected construct (Fig. 8D and E). Based on these results, we conclude that *asd-5* is actually *rsd-6. rsd-6* is known to be engaged in an endogenous gene-silencing pathway that helps maintain genome integrity under unfavorable conditions (43, 44). Thus, our study for the first time identified an interplay between antiviral innate immunity and the mechanism that maintains genome integrity.

### *asd-2* and *asd-9* are required for RNAi targeting Orsay virus.

**FIG 9:**
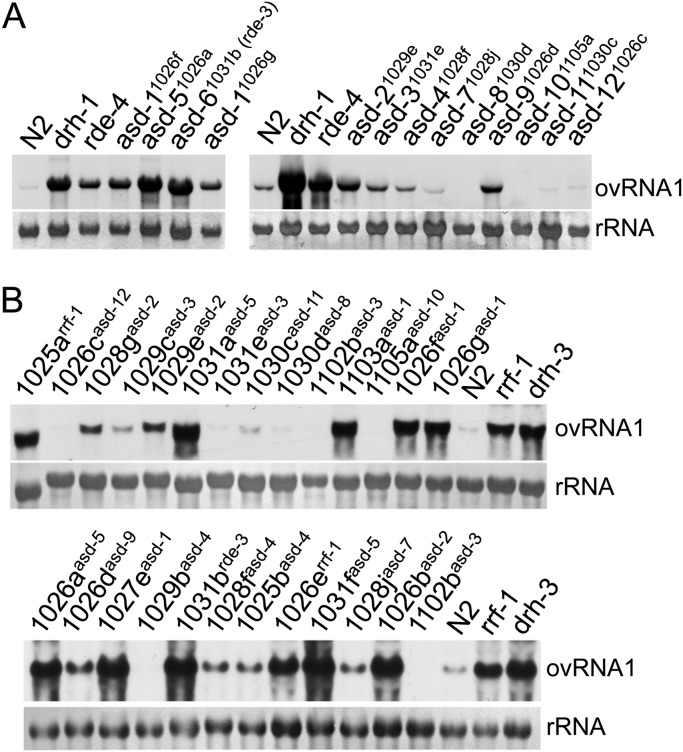
Both *asd-2* and *asd-9* play important roles in antiviral RNAi targeting Orsay virus. (A) Northern blot detection of Orsay virus replication in LR11 worms that carry representative alleles corresponding to each of the candidate genes as indicated. (B) Northern blot detection of Orsay virus replication in candidate mutants isolated from the generic screen. ovRNA1, Orsay virus RNA1.

To find out whether the identified genes are required for antiviral RNAi against Orsay virus, we checked Orsay virus replication in LR11 worms containing the identified alleles. As shown in Fig. 9A, although enhanced Orsay virus replication was detected for alleles corresponding to *asd-1*, *asd-2*, *asd-5* (*rsd-6*), *asd-6* (*rde-3*), and *asd-9*, such an enhancement was not detected for alleles corresponding to *asd-3*, *asd-4*, *asd-7*, *asd-8*, *asd-10*, *asd-11*, and *asd-12*. As a reconfirmation, we checked Orsay virus replication in original mutants isolated from the genetic screen. As shown in Fig. 9B, enhanced viral replication was detected for all alleles corresponding to *asd-1*, *asd-2*, *asd-5*, and *asd-9* but not for any alleles corresponding to *asd-3*, *asd-4*, *asd-7*, *asd-8*, *asd-10*, *asd-11*, and *asd-12*. These results, together with the results shown in Fig. 6, suggest that *asd-2* and *asd-9* are genes that play important roles in natural antiviral defense mediated by RNAi but are dispensable in classical RNAi triggered by artificial dsRNA. Since these two genes cannot be *drh-1*, which should have been excluded during the screen, we believe that both genes are novel components of worm antiviral RNAi.

### *asd-9* and *rsd-6*, but not *asd-2*, are required for the biogenesis or stability of primary viral siRNAs.

**FIG 10:**
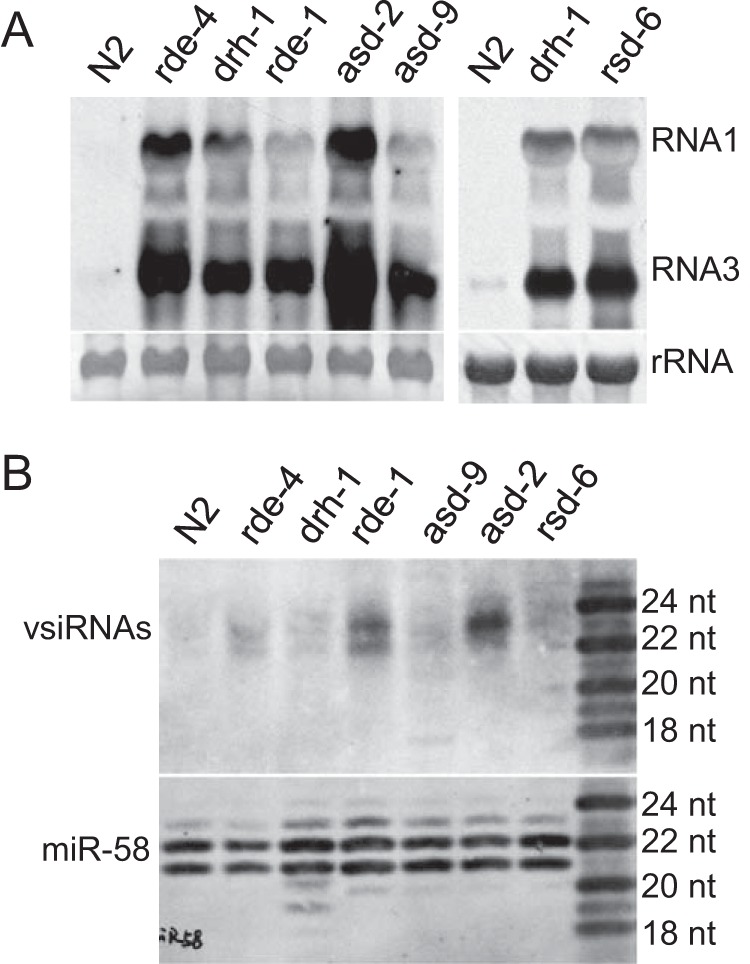
*asd-9* and *rsd-6*, but not *asd-2*, are required for biogenesis or stability of primary viral siRNAs. (A) Accumulation of FR1gfp genomic and subgenomic RNAs in *asd-2* (1029e), *asd-9* (1026d), and *rsd-6* (1026a) after heat induction. The replication of FR1gfp in wild-type N2 worms and *drh-1*, *rde-1*, and *rde-4* mutants was also detected as references. (B) Northern blot detection of FR1gfp-derived primary siRNAs in *asd-2* (1029e), *asd-9* (1026d), *rsd-6* (1026a), and control worm strains. The accumulation of miR-58 was also detected as an equal loading control.

In C. elegans, viral dsRNAs are mainly processed into 23-nucleotide primary vsiRNAs, which can be detected by next-generation sequencing or Northern blotting (6, 8). As an effort to define a role for *asd-2*, *asd-9*, and *rsd-6* in antiviral RNAi, we checked the accumulation of primary vsiRNAs in worm mutants containing *asd-2*, *asd-9*, or *rsd-6* alleles. As references we also detected the accumulation of primary vsiRNAs in *drh-1*, *rde-1*, and *rde-4*. Consistent with previous findings (8, 35), vsiRNAs were detected at high level in *rde-1* mutants but low level in *drh-1* and *rde-4* mutants, although the genomic and subgenomic RNAs of FR1gfp accumulated at comparable levels in these mutants (Fig. 10A and B). Interestingly, we found that primary vsiRNAs were barely detectable in *asd-9* and *rsd-6* mutants but accumulated to high level in *asd-2* mutants (Fig. 10B). When the same blot was stripped and reused for miR-58 detection, we found that miR-58 accumulated at similar levels in these mutants. These results together suggest that *asd-9* and *rsd-6*, but not *asd-2*, are required for the biogenesis or stability of primary viral siRNAs.

## DISCUSSION

Artificial dsRNAs trigger potent silencing of homologous cellular transcripts in diverse organisms, and mechanistic studies of this phenomenon have significantly improved our understanding of antiviral RNAi. However, several lines of evidence suggest that viruses as triggers and targets of RNAi are fundamentally different from the triggers and targets of RNAi in artificial setups. First, viruses often replicate in subcellular compartments, and accordingly virus-produced dsRNAs may be physically isolated from Dicer and cofactors (45). This may explain why antiviral RNAi in C. elegans requires DRH-1, a functional homologue of mammalian virus sensors. Second, the nascent viral transcripts often become rapidly associated with viral structure proteins, such as the coat proteins, and thereby may be protected from Ago-mediated cleavage. The fact that FR1gfp replication triggers potent silencing of homologous cellular transgenes in *drh-1* mutants further suggests that replicating virus is more resistant to RNAi than cellular transcripts, even in the absence of viral structural protein (8). These observations suggest that antiviral RNAi involves more genes than classical RNAi, and these genes can only be identified in genetic screens that utilize replicating virus as a loss-of-RNAi reporter.

Using replicating virus as a reporter for the loss of RNAi activity, we identified 9 candidate genes that appear to be dispensable in classical RNAi. Importantly, we found that among the 9 candidate genes, *asd-2* and *asd-9* are required for antiviral RNAi targeting Orsay virus but appear dispensable for classical RNAi. Since the design of our screen strategy has already excluded *drh-1* as a target of our genetic screen, which is confirmed by *drh-1* coding sequence for our candidates, we believe *asd-2* and *asd-9* are novel antiviral RNAi genes. Mechanistic study of these two genes may allow us to gain further insight into the mechanisms by which viruses are detected and destroyed by the RNAi machinery in C. elegans.

*drh-1* transcriptions are not induced in response to replication of FHV (16). Currently, whether the function of DRH-1 in virus detection undergoes posttranslational regulation remains largely unknown. Recently, Choi and colleagues found that the C-terminal region of RIG-I, which plays a crucial role in viral dsRNA detection, undergoes deacetylation to regulate its activity in virus sensing and that RIG-I deacetylation by HDAC6 (histone deacetylase 6) is critical for viral RNA detection (46). The residue targeted by HDAC6 for deacetylation is within a KWK motif that is conserved in both RIG-I and DRH-1. Since the KWK motif is required for DRH-1 function in antiviral RNAi, it would be interesting to check if DRH-1 also undergoes deacetylation at this particular residue and whether deacetylation of this residue is required for DRH-1 function. Nevertheless, this observation suggests that while its genetic identity awaits further characterization, *asd-9*, which is required for the biogenesis or stability of primary vsiRNAs (Fig. 10B), contributes to viral dsRNA detection by directly or indirectly regulating DRH-1 function.

*rsd-6* was originally identified as one of the genes required for the systemic spreading of RNAi (32). Its role in antiviral RNAi has never been reported. Previously it has been shown that meiotic chromosome disjunction is affected in *rsd-6* mutants under stressful conditions (43). Further study of this phenomenon revealed that *rsd-6* helps maintain genome integrity in stressful environments by maintaining transgenerational inheritance of endogenous siRNA populations that promote genome silencing (44). Consistent with this, we found that all *rsd-6* mutants isolated in our genetic screen produce far fewer progenies at room temperature than wild-type worms. However, our *rsd-6* mutants do not become completely sterile when reared at 25°C (Table 1). Probably other worm genes, such as *rsd-2*, partially compensate for the loss of *rsd-6* function in maintaining genome integrity. The fact that *rsd-6* is required for the biogenesis or stability of primary vsiRNAs (Fig. 10B) suggests that *rsd-6* is required for the production of secondary vsiRNAs whose biogenesis relies on the production and function of primary vsiRNAs. In support of this hypothesis, *rsd-6* was previously found to help maintain the population of a class of endogenous secondary siRNAs that target and silence genes involved in spermatogenesis (44).

Previously we have demonstrated that replicating virus is more resistant to RNAi than cellular transcripts (8), suggesting that the secondary siRNA-mediated amplification mechanism is more important for RNAi to destroy replicating viruses than cellular transcripts. *rde-10*, *rde-11*, and *rsd-2* are 3 genes that are required for the production of secondary siRNAs. Recently, it has been demonstrated that worm mutants corresponding to these genes remain sensitive to high dosage of dsRNA triggers (33, 47), suggesting that a secondary siRNA-mediated gene-silencing effect would not be detected in a classical RNAi screen where the dosage of the dsRNA trigger is high. In fact, we also observed that *rrf-1* mutants are sensitive to RNAi triggered by ingestion or injection of dsRNA (Fig. 5F and data not shown). These observations together suggest that some of our candidate genes that are dispensable for classical RNAi mainly contribute to the biogenesis and/or function of secondary siRNA in antiviral RNAi. We speculate that *asd-2* is such an antiviral RNAi gene. Although its function is not required for the biogenesis or stability of primary vsiRNAs (Fig. 10B), *asd-2* may contribute to the biogenesis and/or function of secondary vsiRNAs in antiviral RNAi.

Surprisingly, 7 of the candidate genes that are dispensable for classical RNAi do not confer resistance to intestine-infecting Orsay virus (Fig. 9). To rule out the possibility that these genes regulate the transcription of the FR1gfp replicon transgene, thereby suppressing the replication of FR1gfp, we tested the antiviral activity of candidate genes *asd-4* and *asd-7* using worms that contain an Orsay virus replicon transgene driven by a heat-inducible promoter. After heat induction we did not observe significant increase in Orsay virus replication in *asd-4* or *asd-7* mutants compared to wild-type worms (F. Meng, unpublished data). Currently it remains possible that those genes mainly function in nonintestinal cells to confer virus-specific silencing.

Its short life span and hermaphroditic lifestyle make C. elegans a powerful system for gene identification through forward genetic screen. However, large-scale forward genetic screen in the C. elegans system is still a time-consuming and labor-intensive process, mainly because many of the target genes are repeatedly picked up during the screen, and consequently much effort will be wasted in characterizing the redundant alleles. By introducing extra copies of known antiviral RNAi genes into the reporter worm strain, we were able to successfully reject alleles corresponding to those genes during genetic screen. Since codelivery of multiple transgenes into C. elegans through gonad microinjection can be done in laboratories with basic microinjection facilities, this strategy can be easily adopted for identification of novel genes involved in other biological pathways. The small body size and short life cycle of C. elegans suggest that gene discovery in the C. elegans system can be easily scaled up to screen a very large number of mutagenized worms, even in laboratories with limited resources. Thus, our approach combined with a mapping-by-sequencing strategy will allow for rapid identification of functionally redundant genes and genes with low mutation rates.

## MATERIALS AND METHODS

### Genetics.

N2, a Bristol isolate of wild-type C. elegans, was used as a reference strain in this study. Other N2-derived mutants used in this study are *rde-1*(*ne300*), *rde-4*(*ne337*), *rsd-2*(*pk3307*), *drh-1*(*tm329*), *rrf-1*(*ok589*), and *drh-3*(*ne4253*). The genotypes of *rde-1*, *rde-4*, and *rsd-2* mutants were confirmed by *skn-1* feeding RNAi together with DNA sequencing. The genotype for *drh-1* and *rrf-1* mutants was confirmed by PCR. The *drh-3* mutants are sterile at 25°C and thus are selected based on their temperature sensitivity.

### Plasmid constructs and transgenic worms.

All plasmid constructs created for constitutive expression of the antiviral RNAi genes were developed using the PD51 vector as the backbone (8). The development of Psur-5::drh-1 and Psur-5::rsd-2 has been described previously (8, 31). Plasmids Psur-5::rde-1 and Psur-5::rde-4 were created by inserting the *rde-1* and *rde-4* coding sequences, amplified through reverse transcription-PCR (RT-PCR), into PD51. The resulting constructs were confirmed by DNA sequencing. The development of FR1gfp replicon was described previously (16).

### RNAi assay.

All feeding RNAi plates were seeded with bacterial strains, derived from HT115, engineered to express double-stranded RNA corresponding to the target genes. IPTG (isopropyl-β-d-thiogalactopyranoside) at a final concentration of 5 mM was used to induce the production of dsRNA in HT115. Worms at the L1 or L2 larval stage were transferred onto freshly prepared feeding RNAi plates. NGM plates containing OP50 food were used as controls in each experiment. To prepare dsRNA for microinjection, equal amounts of plus- and minus-stranded RNA molecules, synthesized through *in vitro* transcription, were combined in ultrapure H_2_O, denatured in boiling water for 5 min, and annealed at room temperature. The RNAi phenotypes were recorded for progeny worms collected between 8 and 32 h after microinjection.

### Genetic screen.

Our genetic screen was carried out by following an established protocol, with some minor modifications. We treated synchronized L4 worms with 47 μM EMS for 6 h to introduce random mutations into the worm genome. The F1 progenies were then bleached to collect eggs for genetic screening. All F2 worms that produced green fluorescence after heat induction were picked up and propagated for reconfirmation. All confirmed mutants were then labeled based on the day they were identified and subjected to further characterization, such as temperature sensitivity and genetic complementation tests.

### Orsay virus inoculum preparation and infection of C. elegans.

Orsay virus was maintained in worm strain JU1580 by following a protocol described previously (14). To prepare infectious Orsay virus filtrate, infected JU1580 worms were washed off of slightly starved NGM plates using sterilized water. After a quick low-speed spin to pellet the worms, the supernatant was filtered through 0.22-μm filter units and the filtrate was mixed with OP50 culture for NGM plate seeding.

### RNA gel blot analysis for viral RNA detection.

The FR1gfp replication in worms containing the FR1gfp transgene was induced by incubating the transgenic worms at 33°C for 3 h. The total worm RNA was then extracted with TRI Reagent by following the manufacturer's instructions (Sigma-Aldrich, Inc.). The FR1gfp transcripts were then detected using a protocol described previously (38). The detection of Orsay virus replication was done using the same protocol, except that the cDNA probe was RT-PCR amplified from the RNA1 of Orsay virus. The primary vsiRNAs were detected using homolog-labeled oligonucleotide probes as described previously (38).

### Candidate allele mapping through whole-genome sequencing.

Hermaphrodites carrying the candidate allele were crossed to N2 males that carry an FR1gfp transgene. Fifty F2 worms that carry homozygous candidate alleles, namely, the mutant pool, and 50 F2 worms that do not carry the candidate allele, namely, the wild-type pool, were then singled out and propagated on 60-mm petri dishes for genomic DNA preparation. After the genomic DNA was extracted for each F2 strain using a Qiagen DNeasy blood and tissue kit, the genomic DNA was combined at equal amounts to produce a mutant pool and wild-type pool. To construct libraries for whole-genome sequencing, the combined genomic DNA was sheared using a Biorupter (15 s on, 90 s off, for 9 cycles). The PCR-free TruSeq DNA kit (Illumina) then was used to construct libraries for deep sequencing. Six of the genomic DNA libraries were sequenced on one lane for 161 cycles from one end of the fragments on an Illumina HiSeq 2500 platform. The reads are 160 nucleotides in length. Processing of the Illumina reads, mapping, and variation identification were performed by following the workflow illustrated in Fig. 7. The wild-type N2 reference genome and annotation used in our study was WormBase release WS235 (http://www.wormbase.org).

### Imaging microscopy.

GFP and mCherry fluorescence images were recorded using a Nikon p7000 digital camera mounted on a Nikon SMZ1500 microscope.

## References

[1] Ding SW, Voinnet O 2007 Antiviral immunity directed by small RNAs. Cell 130:413–426. doi:10.1016/j.cell.2007.07.039. 17693253 PMC2703654

[2] Roghiyh Aliyari S-WD 2009 RNA-based viral immunity initiated by the Dicer family of host immune receptors. Immunol Rev 227:176–188. doi:10.1111/j.1600-065X.2008.00722.x. 19120484 PMC2676720

[3] Parker GS, Eckert DM, Bass BL 2006 RDE-4 preferentially binds long dsRNA and its dimerization is necessary for cleavage of dsRNA to siRNA. RNA 12:807–818. doi:10.1261/rna.2338706. 16603715 PMC1440910

[4] Schott DH, Cureton DK, Whelan SP, Hunter CP 2005 An antiviral role for the RNA interference machinery in Caenorhabditis elegans. Proc Natl Acad Sci U S A 102:18420–18424. doi:10.1073/pnas.0507123102. 16339901 PMC1317933

[5] Tabara H, Yigit E, Siomi H, Mello CC 2002 The dsRNA binding protein RDE-4 interacts with RDE-1, DCR-1, and a DExH-box helicase to direct RNAi in C. elegans. Cell 109:861–871. 12110183 10.1016/s0092-8674(02)00793-6

[6] Ashe A, Bélicard T, Le Pen J, Sarkies P, Frézal L, Lehrbach NJ, Félix M-A, Miska EA, Weigel D 2013 A deletion polymorphism in the Caenorhabditis elegans RIG-I homolog disables viral RNA dicing and antiviral immunity. Elife 2:e00994. doi:10.7554/eLife.00994. 24137537 PMC3793227

[7] Garcia-Ruiz H, Takeda A, Chapman EJ, Sullivan CM, Fahlgren N, Brempelis KJ, Carrington JC 2010 Arabidopsis RNA-dependent RNA polymerases and Dicer-like proteins in antiviral defense and small interfering RNA biogenesis during turnip mosaic virus infection. Plant Cell 22:481–496. 20190077 10.1105/tpc.109.073056 PMC2845422

[8] Guo X, Zhang R, Wang J, Ding S-W, Lu R 2013 Homologous RIG-I-like helicase proteins direct RNAi-mediated antiviral immunity in C. elegans by distinct mechanisms. Proc Natl Acad Sci U S A 110:16085–16090. doi:10.1073/pnas.1307453110. 24043766 PMC3791698

[9] Wang X-B, Wu Q, Ito T, Cillo F, Li W-X, Chen X, Yu J-L, Ding S-W 2010 RNAi-mediated viral immunity requires amplification of virus-derived siRNAs in Arabidopsis thaliana. Proc Natl Acad Sci U S A 107:484–489. doi:10.1073/pnas.0904086107. 19966292 PMC2806737

[10] Li Y, Lu J, Han Y, Fan X, Ding S-W 2013 RNA interference functions as an antiviral immunity mechanism in mammals. Science 342:231–234. doi:10.1126/science.1241911. 24115437 PMC3875315

[11] Maillard PV, Ciaudo C, Marchais A, Li Y, Jay F, Ding SW, Voinnet O 2013 Antiviral RNA interference in mammalian cells. Science 342:235–238. doi:10.1126/science.1241930. 24115438 PMC3853215

[12] Diaz-Pendon JA, Ding SW 2008 Direct and indirect roles of viral suppressors of RNA silencing in pathogenesis. Annu Rev Phytopathol 46:303–326. doi:10.1146/annurev.phyto.46.081407.104746. 18680427

[13] Ding S-W, Lu R 2011 Virus-derived siRNAs and piRNAs in immunity and pathogenesis. Curr Opin Virol 1:1–12. doi:10.1016/j.coviro.2011.04.001. PMC3237678 22180767

[14] Félix M-A, Ashe A, Piffaretti J, Wu G, Nuez I, Bélicard T, Jiang Y, Zhao G, Franz CJ, Goldstein LD, Sanroman M, Miska EA, Wang D 2011 Natural and experimental infection of Caenorhabditis nematodes by novel viruses related to nodaviruses. PLoS Biol 9:e1000586. doi:10.1371/journal.pbio.1000586. 21283608 PMC3026760

[15] Ketting RF, Fischer SE, Bernstein E, Sijen T, Hannon GJ, Plasterk RH 2001 Dicer functions in RNA interference and in synthesis of small RNA involved in developmental timing in C. elegans. Genes Dev 15:2654–2659. doi:10.1101/gad.927801. 11641272 PMC312808

[16] Lu R, Yigit E, Li WX, Ding SW 2009 An RIG-I-like RNA helicase mediates antiviral RNAi downstream of viral siRNA biogenesis in Caenorhabditis elegans. PLoS Pathog 5:e1000286. doi:10.1371/journal.ppat.1000286. 19197349 PMC2629121

[17] Lu R, Maduro M, Li F, Li HW, Broitman-Maduro G, Li WX, Ding SW 2005 Animal virus replication and RNAi-mediated antiviral silencing in Caenorhabditis elegans. Nature 436:1040–1043. doi:10.1038/nature03870. 16107851 PMC1388260

[18] Wilkins C, Dishongh R, Moore SC, Whitt MA, Chow M, Machaca K 2005 RNA interference is an antiviral defence mechanism in Caenorhabditis elegans. Nature 436:1044–1047. doi:10.1038/nature03957. 16107852

[19] Yigit E, Batista PJ, Bei Y, Pang KM, Chen CC, Tolia NH, Joshua-Tor L, Mitani S, Simard MJ, Mello CC 2006 Analysis of the C. elegans Argonaute family reveals that distinct Argonautes act sequentially during RNAi. Cell 127:747–757. doi:10.1016/j.cell.2006.09.033. 17110334

[20] Steiner FA, Okihara KL, Hoogstrate SW, Sijen T, Ketting RF 2009 RDE-1 slicer activity is required only for passenger-strand cleavage during RNAi in Caenorhabditis elegans. Nat Struct Mol Biol 16:207–211. doi:10.1038/nsmb.1541. 19151723

[21] Aoki K, Moriguchi H, Yoshioka T, Okawa K, Tabara H 2007 In vitro analyses of the production and activity of secondary small interfering RNAs in C. elegans. EMBO J 26:5007–5019. doi:10.1038/sj.emboj.7601910. 18007599 PMC2140100

[22] Pak J, Fire A 2007 Distinct populations of primary and secondary effectors during RNAi in C. elegans. Science 315:241–244. doi:10.1126/science.1132839. 17124291

[23] Sijen T, Steiner FA, Thijssen KL, Plasterk RH 2007 Secondary siRNAs result from unprimed RNA synthesis and form a distinct class. Science 315:244–247. doi:10.1126/science.1136699. 17158288

[24] Gent JI, Lamm AT, Pavelec DM, Maniar JM, Parameswaran P, Tao L, Kennedy S, Fire AZ 2010 Distinct phases of siRNA synthesis in an endogenous RNAi pathway in C. elegans Soma. Mol Cell 37:679–689. doi:10.1016/j.molcel.2010.01.012. 20116306 PMC2838994

[25] Zou J, Chang M, Nie P, Secombes CJ 2009 Origin and evolution of the RIG-I like RNA helicase gene family. BMC Evol Biol 9:85. doi:10.1186/1471-2148-9-85. 19400936 PMC2686710

[26] Jiang F, Ramanathan A, Miller MT, Tang G-Q, Gale M, Patel SS, Marcotrigiano J 2011 Structural basis of RNA recognition and activation by innate immune receptor RIG-I. Nature 479:423–427. doi:10.1038/nature10537. 21947008 PMC3430514

[27] Jiang Q-X, Chen ZJ 2012 Structural insights into the activation of RIG-I, a nanosensor for viral RNAs. EMBO Rep 13:7–8. doi:10.1038/embor.2011.239. PMC3246263 22157887

[28] Kowalinski E, Lunardi T, Andrew McCarthy A, Louber J, Brunel J, Grigorov B, Gerlier D, Cusack S 2011 Structural basis for the activation of innate immune pattern-recognition receptor RIG-I by viral RNA. Cell 147:423–435. doi:10.1016/j.cell.2011.09.039. 22000019

[29] Luo D, Ding SC, Vela A, Kohlway A, Lindenbach BD, Pyle AM 2011 Structural insights into RNA recognition by RIG-I. Cell 147:409–422. doi:10.1016/j.cell.2011.09.023. 22000018 PMC3222294

[30] Duchaine TF, Wohlschlegel JA, Kennedy S, Bei Y, Conte D Jr, Pang K, Brownell DR, Harding S, Mitani S, Ruvkun G, Yates JR III, Mello CC 2006 Functional proteomics reveals the biochemical niche of C. elegans DCR-1 in multiple small-RNA-mediated pathways. Cell 124:343–354. doi:10.1016/j.cell.2005.11.036. 16439208

[31] Guo X, Zhang R, Wang J, Lu R 2013 Antiviral RNA silencing initiated in the absence of RDE-4, a double-stranded RNA binding protein, in Caenorhabditis elegans. J Virol 87:10721–10729. doi:10.1128/JVI.01305-13. 23885080 PMC3807410

[32] Tijsterman M, May RC, Simmer F, Okihara KL, Plasterk RH 2004 Genes required for systemic RNA interference in Caenorhabditis elegans. Curr Biol 14:111–116. doi:10.1016/j.cub.2003.12.029. 14738731

[33] Zhang C, Montgomery TA, Fischer SEJ, Garcia SMDA, Riedel CG, Fahlgren N, Sullivan CM, Carrington JC, Ruvkun G 2012 The Caenorhabditis elegans RDE-10/RDE-11 complex regulates RNAi by promoting secondary siRNA amplification. Curr Biol 22:881–890. doi:10.1016/j.cub.2012.04.011. 22542102 PMC3371361

[34] Shirayama M, Stanney W III, Gu W, Seth M, Mello C 2014 The Vasa homolog RDE-12 engages target mRNA and multiple argonaute proteins to promote RNAi in C. elegans. Curr Biol 24:845–851. doi:10.1016/j.cub.2014.03.008. 24684931 PMC4017897

[35] Coffman SR, Lu J, Guo X, Zhong J, Jiang H, Broitman-Maduro G, Li W-X, Lu R, Maduro M, Ding S-W 2017 Caenorhabditis elegans RIG-I homolog mediates antiviral RNA interference downstream of Dicer-dependent biogenesis of viral small interfering RNAs. mBio 8:e00264-17. 28325765 10.1128/mBio.00264-17 PMC5362034

[36] Jiang H, Franz CJ, Wang D 2014 Engineering recombinant Orsay virus directly in the metazoan host C. elegans. J Virol 88:11774–11781. doi:10.1128/JVI.01630-14. 25078701 PMC4178717

[37] Guo X, Lu R 2013 Characterization of virus-encoded RNAi suppressors in Caenorhabditis elegans. J Virol 87:5414–5423. doi:10.1128/JVI.00148-13. 23468484 PMC3648182

[38] Guo X, Li W-X, Lu R 2012 Silencing of host genes directed by virus-derived short interfering RNAs in *Caenorhabditis elegans*. J Virol 86:11645–11653. doi:10.1128/JVI.01501-12. 22896621 PMC3486301

[39] Weinheimer I, Jiu Y, Rajamäki M-L, Matilainen O, Kallijärvi J, Cuellar WJ, Lu R, Saarma M, Holmberg CI, Jäntti J, Valkonen JPT 2015 Suppression of RNAi by dsRNA-degrading RNaseIII enzymes of viruses in animals and plants. PLoS Pathog 11:e1004711. doi:10.1371/journal.ppat.1004711. 25747942 PMC4352025

[40] Mello CC, Kramer JM, Stinchcomb D, Ambros V 1991 Efficient gene-transfer in C. elegans–extrachromosomal maintenance and integration of transforming sequences. EMBO J 10:3959–3970. 1935914 10.1002/j.1460-2075.1991.tb04966.x PMC453137

[41] Sijen T, Fleenor J, Simmer F, Thijssen KL, Parrish S, Timmons L, Plasterk RH, Fire A 2001 On the role of RNA amplification in dsRNA-triggered gene silencing. Cell 107:465–476. doi:10.1016/S0092-8674(01)00576-1. 11719187

[42] Tabara H, Sarkissian M, Kelly WG, Fleenor J, Grishok A, Timmons L, Fire A, Mello CC 1999 The rde-1 gene, RNA interference and transposon silencing in C. elegans. Cell 99:123–132. 10535731 10.1016/s0092-8674(00)81644-x

[43] Han W, Sundaram P, Kenjale H, Grantham J, Timmons L 2008 The Caenorhabditis elegans rsd-2 and rsd-6 genes are required for chromosome functions during exposure to unfavorable environments. Genetics 178:1875–1893. doi:10.1534/genetics.107.085472. 18430922 PMC2323783

[44] Sakaguchi A, Sarkies P, Simon M, Doebley A-L, Goldstein LD, Hedges A, Ikegami K, Alvares SM, Yang L, LaRocque JR, Hall J, Miska EA, Ahmed S 2014 Caenorhabditis elegans RSD-2 and RSD-6 promote germ cell immortality by maintaining small interfering RNA populations. Proc Natl Acad Sci U S A 111:E4323–E4331. doi:10.1073/pnas.1406131111. 25258416 PMC4205660

[45] den Boon JA, Diaz A, Ahlquist P 2010 Cytoplasmic viral replication complexes. Cell Host Microbe 8:77–85. doi:10.1016/j.chom.2010.06.010. 20638644 PMC2921950

[46] Choi SJ, Lee HC, Kim JH, Park SY, Kim TH, Lee WK, Jang DJ, Yoon JE, Choi YI, Kim S, Ma J, Kim CJ, Yao TP, Jung JU, Lee JY, Lee JS 2016 HDAC6 regulates cellular viral RNA sensing by deacetylation of RIG-I. EMBO J 35:429–442. 26746851 10.15252/embj.201592586 PMC4755110

[47] Yang H, Zhang Y, Vallandingham J, Li H, Florens L, Mak HY 2012 The RDE-10/RDE-11 complex triggers RNAi-induced mRNA degradation by association with target mRNA in C. elegans. Genes Dev 26:846–856. doi:10.1101/gad.180679.111. 22508728 PMC3337458

